# Skin of colour dermatology education in US primary care residency programmes: a nationwide cross-sectional survey of programme directors

**DOI:** 10.1093/skinhd/vzae001

**Published:** 2025-01-23

**Authors:** Lauren C LaMonica, Thomas Hester, Reinie Thomas, Frank Wang

**Affiliations:** Department of Dermatology, University of Michigan, Ann Arbor, MI, USA; Department of Dermatology, University of Michigan, Ann Arbor, MI, USA; Department of Dermatology, University of Michigan, Ann Arbor, MI, USA; Department of Dermatology, University of Michigan, Ann Arbor, MI, USA

## Abstract

**Background:**

Physicians-in-training report inadequate education in skin of colour (SOC) dermatology during residency. Although dermatology programmes have made progress in teaching SOC dermatology, the status of SOC dermatology education in primary care residency programmes remains unclear.

**Objectives:**

To characterize SOC didactic and clinical training opportunities available to primary care residents, laying the groundwork for future curriculum development of SOC dermatology.

**Methods:**

This cross-sectional study consisted of a nationwide 16-question survey disseminated by email between October 2022 and February 2023 to US primary care residency programmes identified using the Accreditation Council for Graduate Medical Education (ACGME) database.

**Results:**

Of responding programmes (*n* = 49/1224, 4.0%), 29/49 offered SOC didactic training, most often through integration of content within general dermatology lectures (*n* = 29/49, 59.2%) and board review sessions (*n* = 13/49, 26.5%). Over half of programmes (*n* = 35/49, 71.4%) offered SOC clinical training through rotation in a general dermatology clinic (*n* = 33/49, 67.3%) and skin-related chief concerns in primary care clinics (*n* = 29/49, 59.2%). Programmes with directors indicating that they planned to incorporate SOC education into future curricula (*n* = 20/49, 40.8%) were more likely to already have SOC didactic and clinical training opportunities (*P* = 0.01 and *P* = 0.02, respectively). Regarding future directions, programme directors were most interested in integrating SOC topics within dermatology lectures (*n* = 31/49, 63.3%); identifying an expert (*n* = 31/49, 63.3%) and allocating lecture time (*n* = 10/49, 20.4%) were the most frequently cited barriers.

**Conclusions:**

Some primary care programmes provide SOC dermatology didactic and clinical training opportunities, which are influenced by programme directors’ willingness to incorporate such training into curricula, and present opportunities for dermatologists to educate primary care residents.

## Teaser

Physicians-in-training report inadequate education in skin of colour (SOC) dermatology during residency. Although dermatology programmes have made progress in teaching SOC dermatology, the status of SOC dermatology education in primary care residency programmes remains unclear. This study aims to characterize the SOC didactic and clinical training opportunities available to primary care residents, laying the groundwork for future curriculum development.

**What is already known about this topic?**

- Physicians-in-training report inadequate education in skin of colour (SOC) dermatology during residency.
- Although dermatology residency programmes have made substantial progress in teaching SOC dermatology, the status of SOC dermatology education in primary care residency programmes remains unclear.

**What does this study add?**

- This study provides data on SOC dermatology didactic and clinical training opportunities available in primary care residency programmes, programme directors’ willingness to incorporate SOC dermatology education into the curriculum, specific types of curricular offerings they would be willing to implement and barriers to implementation.
- The results of this study identify meaningful opportunities for dermatologists to educate primary care residents by teaching SOC dermatology.

By 2050, individuals with skin of colour (SOC) will account for over half (52%) of the US population.^1,2^ Despite this projection, SOC dermatology has historically been under-­represented in medical training.^3^ Indeed, physicians-in-­training report inadequate education in SOC dermatology during residency, which impacts confidence and delivery of care for patients with SOC.^4–6^ In a recent study, more than 90% of internal medicine residents reported insufficient training and low confidence in identifying SOC dermatology educational resources for patient care.^7^ In an evaluation of experiences of Black patients treated in an SOC dermatology clinic compared with a general dermatology clinic, specialized knowledge of the care of Black skin was identified as an important predictor of positive ratings of care.^8^

Although dermatology residency programmes have made substantial progress in teaching SOC dermatology,^5^ the status of SOC dermatology education in primary care residency programmes remains unclear. This study aims to characterize the SOC didactic and clinical training opportunities available to primary care residents, laying the groundwork for future curriculum development.

## Materials and methods

An originally designed Qualtrics (Provo, UT, USA) survey was sent by email to 1224 US primary care residency programmes identified using the Accreditation Council for Graduate Medical Education (ACGME) database (Appendix S1; see Supporting Information). It consisted of 16 questions about programme demographics, availability of SOC dermatology didactic and clinical training opportunities, programme directors’ willingness to incorporate SOC dermatology education into the curriculum, specific types of curricular offerings they would be willing to implement, and barriers to implementation. Responses were collected from October 2022 to February 2023.

Only respondents who completed all survey questions were included for analysis. The distribution of variables was analysed by χ^2^ statistics (categorical variables) using R software v1.2 (SAS Institute, Cary, NC, USA). *P*-values (two-sided) < 0.05 were considered statistically significant.

## Results

In total, 49 residency programmes (34 family medicine, 15 internal medicine) completed the survey. Most indicated that their primary training site was a community-based, university-affiliated hospital (*n* = 21/49, 42.9%) in an urban setting (*n* = 30/49, 61.2%). Geographically, most were located in the southern states of the USA (*n* = 17/49, 34.7%, Table 1). Most programmes (*n* = 35/49, 71.4%) did not have an affiliated dermatology residency programme at the primary training site; however, this association did not impact the availability of SOC didactic (*P* = 1.00) and clinical (*P* = 0.48) training opportunities.

**Table 1:** Demographic data of survey respondents (*n* = 49)

Variable	*n* (%)
Residency training programme type	
Family medicine	34 (69.4)
Internal medicine	13 (26.5)
Internal medicine, primary care track	1 (2.0)
Other^a^	1 (2.0)
Primary training site	
Community-based, university-affiliated hospital	21 (42.9)
University-based academic medical centre	13 (26.5)
Community-based hospital	11 (22.4)
Military hospital	2 (4.1)
Other^b^	2 (4.1)
Programme setting	
Urban	30 (61.2)
Suburban	13 (26.5)
Rural	6 (12.2)
Programme location^c^	
South	17 (34.7)
Midwest	15 (30.6)
West	10 (20.4)
Northeast	7 (14.3)
Dermatology residency offered at training site	
No	35 (71.4)
Yes	14 (28.6)

^a^Other (free-text response): both internal medicine and internal medicine, primary care track. ^b^Other (free-text response): tribally affiliated. ^c^USA.

Regarding didactic opportunities, 59.2% of programmes (*n* = 29/49) offered education in diagnosing/treating SOC dermatological conditions, most often through integration of content within general dermatology lectures (*n* = 29/49, 59.2%), board review sessions (*n* = 13/49, 26.5%) and online self-directed modules (*n* = 11/49, 22.4%, Table 2). When SOC didactic material was integrated within general dermatology lectures, 48.3% (*n* = 14/49) were led by dermatology faculty.

**Table 2:** Skin of colour (SOC) didactic and clinical training opportunities offered within primary care programmes (*n* = 49)

Variable	*n* (%)
SOC didactic training	
Yes	29 (59.2)
No	20 (40.8)
SOC dermatology didactic training opportunities offered	
Exposure to SOC dermatology topics, integrated within other dermatology lectures	29 (59.2)
Exposure through board review sessions	13 (26.5)
Online self-directed training modules	11 (22.4)
Exposure through case-based presentations, including Morbidity & Mortality conference	9 (18.4)
Exposure through grand rounds presentation	7 (14.3)
Exposure to SOC dermatology topics, integrated within nondermatology lectures	6 (12.2)
Opportunities to attend regional/national conferences that teach SOC dermatology	5 (10.2)
One dedicated lecture on SOC dermatology	4 (8.2)
Invited/guest lecturers on SOC dermatology from outside institution	3 (6.1)
Exposure through journal clubs	3 (6.1)
Two or more dedicated lectures on SOC dermatology	2 (4.1)
Other^a^	1 (2.0)
No formal didactic training	10 (20.4)
Session leads for exposure to SOC dermatology topics, when integrated within other dermatology lectures (*n* = 29)	
Dermatology attending	14 (48.3)
Nondermatology attending	11 (37.9)
Nondermatology resident	2 (6.9)
Dermatology resident	1 (3.4)
Other^b^	1 (3.4)
SOC clinical training	
Yes	35 (71.4)
No	14 (28.6)
SOC dermatology clinical training opportunities offered	
Rotation in a general dermatology clinic	33 (67.3)
Skin chief complaints in primary care clinic (experiential learning)	29 (59.2)
Interaction with outpatient dermatology consultants	15 (30.6)
Interaction with inpatient dermatology consultants	11 (22.4)
Rotation in a SOC, multiethnic or multicultural dermatology clinic	7 (14.3)
Rotation on an inpatient dermatology service	5 (10.2)
Other^c^	2 (4.1)
No formal clinical training	8 (16.3)

^a^Other (free-text response): clinic sessions with dermatology attending. ^b^Other (free-text response): nondermatology attendings and residents. ^c^Other (free-text response): primary care track-specific dermatology clinic.

In addition to didactic teaching, over half of programmes (*n* = 35/49, 71.4%) offered SOC clinical training opportunities, most often through rotation in a general dermatology clinic (*n* = 33/49, 67.3%), experiential learning through skin-related chief concerns in primary care clinics (*n* = 29/49, 59.2%) and interaction with outpatient dermatology consultants (*n* = 15/49, 30.6%, Table 2).

In terms of future directions, programmes with directors who indicated they definitely planned to incorporate SOC education into their curricula (*n* = 20/49, 40.8%, Table 3) were more likely to already offer SOC dermatology didactic and clinical training opportunities (*P* = 0.01 and *P* = 0.02, respectively) (Table 3). Overall, programme directors were most interested in the following training opportunities: integrating SOC topics within dermatology lectures (*n* = 31/49, 63.3%), one dedicated lecture in SOC dermatology (*n* = 20/49, 40.8%) and exposure to SOC dermatology topics integrated within nondermatology lectures (*n* = 12/49, 24.5%, Table 4). Identifying an expert lecturer (*n* = 31/49, 63.3%) and allocating lecture time (*n* = 10/49, 20.4%) were the most frequently cited barriers to incorporating SOC dermatology training into curricula.

**Table 3:** Availability of skin of colour (SOC) didactic and clinical training opportunities among programme directors willing to implement SOC curricula

Variable	Total sample (*n* = 49)	Programme director willing to definitely implement SOC curricula (*n* = 20)	Programme director not definitely willing to implement SOC curricula (*n* = 29)	*P*-value^a^
Didactic training offered in diagnosing/treating SOC skin conditions				
Yes	29 (59.2)	16 (80.0)	13 (44.8)	0.01
No	20 (40.8)	4 (20.0)	16 (55.2)	
Clinical training offered in diagnosing/treating SOC skin conditions				
Yes	35 (71.4)	18 (90.0)	17 (58.6)	0.02
No	14 (28.6)	2 (10.0)	12 (41.4)	

Data are presented as n (%) unless otherwise stated.

^a^ *P* value represents χ^2^ test for categorical variables.

**Table 4:** Programme director willingness to implement skin of colour (SOC) dermatology training (*n* = 49)

Variable	*n* (%)
SOC dermatology training opportunities programme directors are most willing to incorporate^a^	
Exposure to SOC dermatology topics, integrated within other dermatology lectures	31 (63.3)
One dedicated lecture on SOC dermatology	20 (40.8)
Exposure to SOC dermatology topics, integrated within nondermatology lectures	12 (24.5)
Online self-directed training modules	12 (24.5)
Two or more dedicated lectures on SOC dermatology	10 (20.4)
Invited/guest lecturer on SOC dermatology from outside institution	9 (18.4)
Rotation in a general dermatology clinic	9 (18.4)
Rotation in a SOC, multiethnic or multicultural dermatology clinic	8 (16.3)
Exposure through grand rounds presentation	4 (8.2)
Exposure through case-based presentations, including Morbidity & Mortality conference	4 (8.2)
Exposure through board review sessions	3 (6.1)
Opportunities to attend regional/national conferences that teach SOC dermatology	2 (4.1)
Rotation on an inpatient dermatology service	1 (2.0)
Exposure through journal clubs	0 (0.0)
Other	0 (0.0)
Most significant barrier to incorporating SOC dermatology training	
Identifying a lecturer/expert	31 (63.3)
Allocating lecture time	10 (20.4)
Insufficient need given hospital demographics	4 (8.2)
Other^b^	3 (6.1)
Resident Interest	1 (2.0)
Not directly relevant to boards preparation	0 (0.0)

^a^Programmes were allowed to select up to three choices for clinical training opportunities they would be most willing to incorporate. ^b^Other (free-text responses): clinical opportunities for nondermatology residents at teaching site; individuals interested in teaching; very low prevalence of individuals with SOC in region.

## Discussion

Despite efforts to include SOC education in dermatology residency curricula,^5,9^ there are limited, if any, data on the status of SOC dermatology education in US primary care programmes, their programme directors’ willingness to implement SOC education and potential barriers to implementing such curricula. Our data provide insights into these questions, and indicate opportunities for dermatologists to assist primary care programme directors in educating their residents.

Among programmes surveyed, over half offered didactic or clinical education in SOC dermatology, which occurred through various methods, most often through integration of content within general dermatology lectures and rotation in a general dermatology clinic. Interestingly, in terms of available SOC didactic or clinical training opportunities, primary care programmes with and without an associated dermatology programme at the primary teaching site did not appear to be impacted differently. In the latter setting, it is possible that SOC dermatology is taught by dermatologists who are at satellite clinics or not affiliated with the programme, or by primary care programmes. Indeed, we found that only half of lectures integrating SOC content were taught by dermatology faculty. Furthermore, regarding future directions, programme directors expressed the greatest interest in developing didactic training in SOC dermatology, particularly through integration of content within general dermatology lectures. These observations indicate that ample opportunities exist for dermatologists, including those from the community or outside institutions, to teach SOC dermatology to primary care residents. Based on programme directors’ preferences, such opportunities may not require the development of new lectures or materials; in fact, modifying pre-existing lectures to include SOC content and images may be a reasonable start.

The most substantial barrier identified by programme directors was access to an expert lecturer or teacher, suggesting a need to increase accessibility of dermatology teachers for SOC education. To address this barrier, it may be helpful to create a national database of dermatology faculty willing to teach SOC dermatology to primary care programmes. Even if unable to deliver lectures, there may be other ways (as indicated in Table 4) that dermatology faculty can teach SOC dermatology to primary care residents. Moreover, dermatology faculty may consider supporting primary care programmes’ efforts to increase SOC content and images in continuing medical education programmes, board exam or maintenance of certification preparation materials, and lectures at academic conferences, in which there remains a disproportionately low representation of SOC content.^10^ For instance, at annual meetings of the American Academy of Dermatology (AAD) between 1996 and 2005, the percentage of teaching sessions incorporating SOC content was only 2%.^11^ Recently, this figure has improved through the efforts of numerous individuals and organizations, including the Diversity Committee of the AAD, Skin of Color Society and others,^10,12,13^ providing examples/models that may be applied to other specialties, including primary care. Furthermore, dermatology faculty may support curricular changes by supervising or mentoring others, such as junior faculty or dermatology residents, to teach primary care residents.

As expected, we found that training opportunities correlated with residency programme directors’ interest in incorporating SOC dermatology into their curricula. Indeed, such programme directors were more likely to have existing SOC educational opportunities at their programme. To help educate primary care residents, dermatology faculty may consider seeking out and collaborating directly with these interested programme directors, providing advice on curricula, rotations, quality resources and methods to integrate SOC with limited lecture time.

To support these efforts, recent advances in machine learning approaches to dermatology education may be leveraged to better understand gaps in existing SOC curricula among primary care residency programmes and facilitate collaboration by dermatologists.^14^ For example, the Skin Tone Analysis for Representation in Educational Materials (STAR-ED) framework, an end-to-end machine learning tool, may be used to assess under-representation of different skin tones in dermatology education materials, including lectures.^14^ Furthermore, dermatologists may consider recommending validated and interactive e-learning or online resources to primary care programmes for learning SOC dermatology, such as the AAD SOC curriculum.^15^ Finally, gamification or game-based strategies, which can be particularly useful for visual learning, may be used by primary care residency programmes to expand current SOC education,^16^ reinforcing or enhancing concepts or skills, such as the detection of skin cancer.

This study has limitations. As with all survey studies, our findings are subject to response and recall bias. To mitigate response bias, data were collected anonymously. As such, an accurate response rate could not be reported, as we did not know how many programme directors viewed or started the survey without completing it. Overall, data were obtained from a relatively small number of programmes. For similar studies conducted in the future, the number of responses may be improved, in part, by distributing the survey through channels beyond email, providing participants with financial remuneration or sending frequent reminders for completion. Additionally, we observed a higher proportion of respondents from the southern states of the USA compared with other geographical regions, which may indicate selection bias; while we did not collect data on the race/ethnicity of the patient populations served by individual residency programmes, it is possible that programmes in the southern states of the USA care for relatively more patients with SOC. Furthermore, the survey was administered to residency programme directors, but not physicians-in-training. Thus, information regarding other methods that residents use to learn SOC dermatology, such as nationally available or self-initiated online teaching modules, are not likely to have been captured.

In conclusion, SOC dermatology training opportunities are influenced by residency programme directors’ willingness to incorporate such training into their curricula. Although some programmes are providing didactic and clinical training opportunities, access to an expert remains a barrier, indicating that dermatologists could collaborate with interested programme directors to facilitate such opportunities. A combination of didactic sessions and experiential clinical learning may benefit primary care residents’ confidence and skill in treating dermatological conditions in patients with SOC.

## Supplementary Material

vzae001_Supplementary_Data

## Data Availability

The data underlying this article will be shared on reasonable request to the corresponding author.
