# Supplementary material for: Skin of colour dermatology education in US primary care residency programmes: a nationwide cross-sectional survey of programme directors
Source: Skin Health Dis. 2025 Jan 23;5(1):9–13. doi: 10.1093/skinhd/vzae001 (PMC11924359; doi:10.1093/skinhd/vzae001)
Supplement: vzae001_Supplementary_Data [file vzae001_supplementary_data.docx]

**SUPPORTING INFORMATION**

**Appendix S1.** Survey Instrument

Q1 Which of the following best describes your residency training program?

o Internal Medicine (1)

o Internal Medicine, Primary Care Track (2)

o Family Medicine (3)

o Other, please specify (4) __________________________________________________

Q2 Which of the following best describes your program's primary training site?

o University-based academic medical center (1)

o Community-based, university-affiliated hospital (2)

o Community-based hospital (3)

o Military hospital (4)

o Other, please specify (5) __________________________________________________

Q3 Which of the following best describes your program's setting?

o Urban (1)

o Suburban (2)

o Rural (3)

Q4 Which of the following best describes your program's location?

o West (AK, AZ, CA, CO, HI, ID, MT, NV, NM, OR, UT, WA, WY) (1)

o Midwest (IA, IL, IN, KS, MO, MI, MN, ND, NE, OH, SD, WI) (2)

o South (AL, AR, DC, DE, FL, GA, KY, LA, MD, MS, NC, OK, SC, TN, TX, WV, VA) (3)

o Northeast (CT, MA, ME, PA, NH, NJ, NY, RI, VT) (4)

Q5 Is your training site also a site of a dermatology residency?

o Yes (1)

o No (2)

Q6 Are residents offered didactic training in diagnosing/treating skin conditions in patients with skin of color?

o Yes (1)

o No (2)

Q7 Select all of the following SOC dermatology didactic training opportunities available to your residents on a yearly basis (check all that apply)

▢ ⊗No formal didactic training (1)

▢ Exposure to SOC dermatology topics, integrated within other dermatology lectures (2)

▢ Exposure to SOC dermatology topics, integrated within non-dermatology lectures (3)

▢ 1 dedicated lecture on SOC dermatology (4)

▢ 2 or more dedicated lectures on SOC dermatology (5)

▢ Invited/guest lecturers on SOC dermatology from outside your institution (6)

▢ Opportunities to attend regional/national conferences that teach SOC dermatology (7)

▢ Exposure through journal clubs (8)

▢ Exposure through boards review sessions (9)

▢ Exposure through grand rounds presentation (10)

▢ Exposure through case-based presentations, including M&M conference (11)

▢ Online self-directed training modules (12)

▢ Other - please specify (13) __________________________________________________

Display This Question:

If Select all of the following SOC dermatology didactic training opportunities available to your res... = 2 or more dedicated lectures on SOC dermatology

Q8 For "2 or more dedicated lectures on SOC dermatology," who teaches these sessions?

o Dermatology attending (1)

o Non-dermatology attending (2)

o Dermatology resident (3)

o Non-dermatology resident (4)

o Other - please specify (5) __________________________________________________

Display This Question:

If Select all of the following SOC dermatology didactic training opportunities available to your res... = 1 dedicated lecture on SOC dermatology

Q9 For "1 dedicated lecture on SOC dermatology," who teaches this session?

o Dermatology attending (1)

o Non-dermatology attending (2)

o Dermatology resident (3)

o Non-dermatology resident (4)

o Other - please specify (5) __________________________________________________

Display This Question:

If Select all of the following SOC dermatology didactic training opportunities available to your res... = Exposure to SOC dermatology topics, integrated within non-dermatology lectures

Q10 For "Exposure to SOC dermatology topics, integrated within non-dermatology lectures," who teaches these sessions?

o Dermatology attending (1)

o Non-dermatology attending (2)

o Dermatology resident (3)

o Non-dermatology resident (4)

o Other - please specify (5) __________________________________________________

Display This Question:

If Select all of the following SOC dermatology didactic training opportunities available to your res... = Exposure to SOC dermatology topics, integrated within other dermatology lectures

Q11 For "Exposure to SOC dermatology topics, integrated within other dermatology lectures," who teaches these sessions?

o Dermatology attending (1)

o Non-dermatology attending (2)

o Dermatology resident (3)

o Non-dermatology resident (4)

o Other -- please specify (5) __________________________________________________

Q12 Are residents offered clinical training in diagnosing/treating skin conditions in patients with SOC?

o Yes (1)

o No (2)

Q13 Select all of the following SOC dermatology clinical training opportunities available to your residents on a yearly basis (check all that apply)

▢ ⊗No formal clinical training (9)

▢ Rotation in a general dermatology clinic (1)

▢ Rotation in a SOC, multiethnic, or multicultural dermatology clinic (2)

▢ Rotation on an inpatient dermatology service (3)

▢ Skin chief complaints in primary care clinic (experiential learning) (4)

▢ Interaction with dermatology consultants while on an outpatient medicine service (e.g., interaction through in-person or telemedicine referrals) (6)

▢ Interaction with dermatology consultants while on an in-patient medicine service (7)

▢ Other -- please specify (8) __________________________________________________

Q14 I plan to incorporate SOC dermatology training into my program's curriculum

o Definitely yes (1)

o Probably yes (2)

o Might or might not (3)

o Probably not (4)

o Definitely not (5)

Q15 Which of the following SOC dermatology training opportunities would you be most willing to incorporate into your curriculum (select up to three)

▢ Exposure to SOC dermatology topics, integrated within other dermatology lectures (1)

▢ Exposure to SOC dermatology topics, integrated within non-dermatology lectures (2)

▢ 1 dedicated lecture on SOC dermatology (3)

▢ 2 or more dedicated lectures on SOC dermatology (4)

▢ Invited/guest lecturers on SOC dermatology from outside your institution (5)

▢ Opportunities to attend regional/national conferences that teach SOC dermatology (6)

▢ Exposure through journal clubs (7)

▢ Exposure through boards review sessions (8)

▢ Exposure through grand rounds presentation (9)

▢ Exposure through case-based presentations, including M&M conference (10)

▢ Online self-directed training modules (11)

▢ Rotation in a general dermatology clinic (14)

▢ Rotation in a SOC, multiethnic or multicultural dermatology clinic (12)

▢ Rotation on an inpatient dermatology service (13)

▢ Other -- please specify (15)

Q16 Which of the following represents the most significant barrier to incorporating SOC dermatology training into your curriculum?

o Allocating lecture time (1)

o Identifying a lecturer/expert (2)

o Insufficient need given hospital demographics (3)

o Not directly relevant to boards preparation (4)

o Resident interest (5)

o Other -- please specify (6)
